# Cervical Metastases Mimicking Ulnar Neuropathy in a 78-Year-Old Man With Advanced Oesophageal Squamous Cell Carcinoma

**DOI:** 10.7759/cureus.110552

**Published:** 2026-06-09

**Authors:** Kushal Panja, Syed O Husain, Shun Lai Thwin, Zenab Tahir, Bushra Jamil

**Affiliations:** 1 General Medicine, North Manchester General Hospital, Manchester University NHS Foundation Trust, Manchester, GBR; 2 Acute Medicine, North Manchester General Hospital, Manchester University NHS Foundation Trust, Manchester, GBR

**Keywords:** cervical spinal metastasis, lower motor neuron lesion, metastatic spinal cord compression, oesophageal squamous cell carcinoma (scc), ulnar nerve neuropathy

## Abstract

Metastatic spinal cord compression (MSCC) can present with subtle or atypical neurological deficits and may be mistaken for peripheral neuropathy. We report the case of a 78-year-old man with recurrent metastatic mid-oesophageal squamous cell carcinoma who was admitted with community-acquired pneumonia and hypoxia.

During his admission, he developed numbness in the fourth and fifth digits of the left hand, which progressed over 48 hours to weakness and clawing of the hand. Neurological examination demonstrated an ulnar-claw posture with severe intrinsic hand weakness, preserved proximal strength, intact cranial nerves, no identifiable sensory level, and no upper motor neuron signs. Apart from weakness predominantly affecting the ulnar nerve distribution, there were no other focal lower motor neuron findings.

Magnetic resonance imaging (MRI) of the whole spine revealed extensive metastatic disease involving the cervical vertebrae (C4-C7), including a pathological fracture at C7, spinal canal stenosis, and left-sided neuroforaminal involvement at C6-C7 corresponding to the patient’s neurological deficits. Given his frailty and limited prognosis, he was managed conservatively with corticosteroids, cervical immobilisation, and palliative radiotherapy.

This case highlights the risk of diagnostic anchoring on peripheral causes and emphasises the importance of early spinal imaging in patients with cancer who present with new focal neurological deficits, even in the absence of pain or upper motor neuron signs.

## Introduction

Metastatic spinal cord compression (MSCC) is an oncological emergency that can result in irreversible neurological disability if not recognised and treated promptly [1,2]. Delays remain common, and outcomes correlate strongly with neurological function at the time of diagnosis [1,2]. Prospective and guideline-based work emphasise that clinicians should act on symptoms (not only classic late signs such as a sensory level) and proceed rapidly to magnetic resonance imaging (MRI) when MSCC is suspected, as MRI is the most accurate test to confirm the diagnosis and localise the level of compression [1,2].

Although back pain and radicular symptoms are typical early features, MSCC can present atypically, and focal upper-limb deficits may be mistaken for peripheral nerve injury [1,2]. Distinguishing peripheral ulnar neuropathy from proximal pathology, such as C8-T1 radiculopathy or cervical metastatic disease, may be clinically challenging and can contribute to diagnostic anchoring [3]. We report a case of advanced oesophageal squamous cell carcinoma (SCC) with cervical metastatic disease presenting as a rapidly progressive 'ulnar-claw' hand, highlighting the importance of urgent spinal imaging in cancer patients with new focal neurological symptoms, even without pain or upper motor neuron signs.

## Case presentation

A 78-year-old man with a background of recurrent SCC of the mid-oesophagus was admitted with community-acquired pneumonia, presenting with shortness of breath, productive cough, and hypoxia. He was treated with intravenous antibiotics and supportive therapy, with gradual clinical improvement.

His oncological history was significant for definitive chemoradiotherapy for mid-oesophageal SCC in 2020 and radiotherapy for oropharyngeal SCC in 2021. Subsequently, he developed metastatic disease involving a right supraclavicular lymph node and pulmonary nodules confirmed on positron emission tomography (PET) imaging. Due to progressive disease despite systemic therapy, chemotherapy and immunotherapy were discontinued, and he was receiving the best supportive oncological care.

During his admission, he reported new numbness affecting the fourth and fifth digits of the left hand. Over the following 48 hours, this progressed to marked weakness and clawing of the left hand. The patient already had long-standing weakness of the right arm due to a previous shoulder injury; therefore, loss of function in the remaining functional upper limb caused significant distress and functional impairment.

Neurological examination demonstrated clawing of the left hand with severe weakness of finger extension, flexion, abduction, and adduction involving the fourth and fifth digits. Wrist extension was reduced (Medical Research Council grade 3/5), while shoulder and elbow power were preserved (5/5). Thumb movements and power were normal. Sensation to light touch was preserved throughout the left upper limb, although proprioception was mildly reduced in the little finger and ulnar half of the ring finger. Reflexes in the affected limb were absent.

There was no neck pain, radicular pain, skin changes, or limb swelling. Cranial nerve examination was normal with no facial asymmetry, visual disturbance, or bulbar involvement. There were no upper motor neuron signs and no cerebellar features, and the lower limbs were neurologically normal. Bladder and bowel function were preserved.

Initial diagnostic considerations included an ulnar nerve palsy, possible paraneoplastic neuropathy, or metastatic involvement of the cervical spine. Metabolic and nutritional causes were considered unlikely, with normal vitamin B12 and folate levels and no biochemical evidence of deficiency. There was no history of trauma, and a left wrist radiograph was unremarkable (Figure 1).

**Figure 1:**
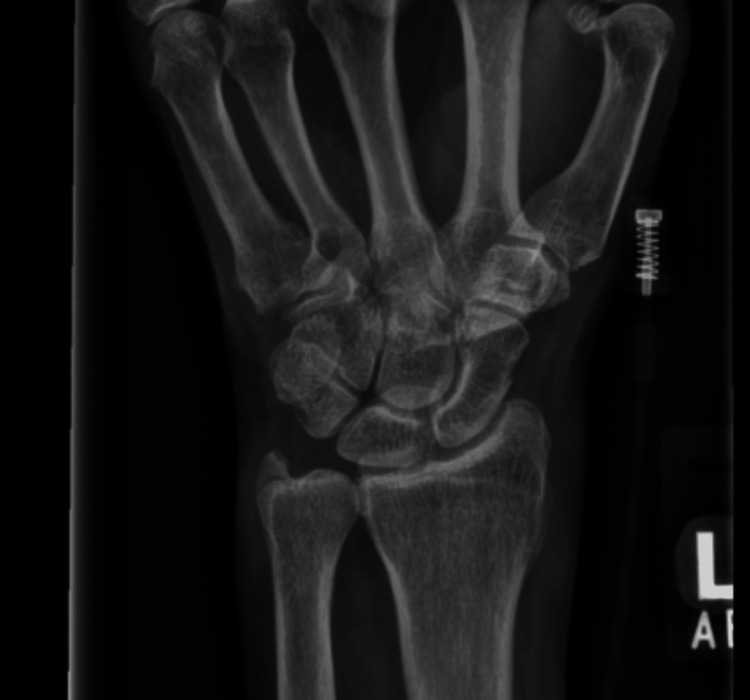
Left wrist radiograph showing no significant osseous or joint abnormality.

Vascular examination of the limb was also normal. Given his known metastatic malignancy and the rapid progression of neurological deficit, urgent MRI of the whole spine was performed following discussion with the oncology team. MRI of the whole spine demonstrated extensive metastatic disease involving the C4-C7 vertebral bodies with a large paravertebral soft-tissue component extending from C4 to T1. There was neuroforaminal involvement, including extension into the left neural foramen at C6-C7 and a pathological fracture at C7 with a posterior cortical bulge resulting in spinal canal stenosis. The imaging findings were consistent with MSCC with associated foraminal involvement, explaining the patient’s focal neurological deficit (Figure 2).

**Figure 2:**
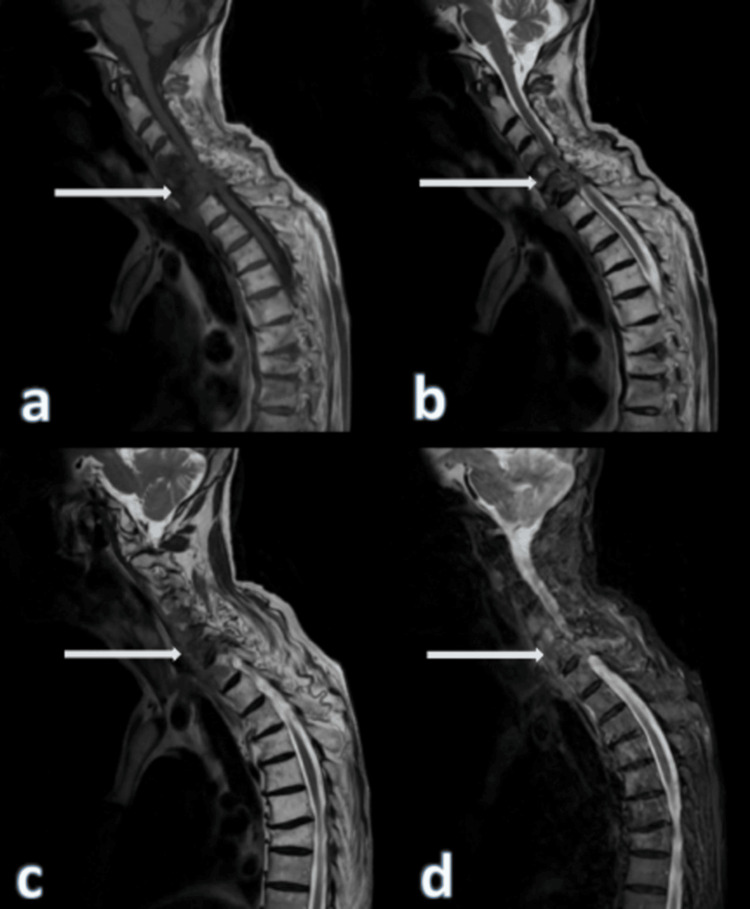
Magnetic resonance imaging (MRI) of the cervical and thoracic spine demonstrating extensive metastatic involvement of the cervical vertebral bodies with associated spinal canal stenosis and neuroforaminal extension. (a) T1-weighted sagittal image. (b) and (c) T2-weighted sagittal images. (d) Short tau inversion recovery (STIR) sagittal image.

Following multidisciplinary discussion, the patient was deemed unsuitable for surgical intervention due to advanced disease, frailty, and limited prognosis. He was managed conservatively with high-dose corticosteroids according to the MSCC protocol, cervical immobilisation, and palliative radiotherapy to the cervical spine.

Despite radiotherapy, there was no significant neurological recovery in the left hand, although further neurological progression was not observed. His overall functional status continued to decline in the context of advanced metastatic disease, and care was subsequently focused on supportive and palliative measures.

## Discussion

MSCC is a recognised oncological emergency that can lead to irreversible neurological deficits if not identified and treated promptly [1,2]. Previous prospective work examining delays in diagnosis has shown that clinicians often wait for classical objective neurological signs before pursuing imaging, despite the fact that early symptoms such as new limb weakness or sensory changes may precede these findings [1]. Current clinical guidance, therefore, emphasises that new neurological symptoms in patients with known malignancy should prompt urgent evaluation and rapid access to MRI, which remains the gold standard diagnostic modality for detecting MSCC and defining the anatomical level of compression [2].

This case illustrates how MSCC may present with an atypical neurological pattern that closely resembles a peripheral nerve palsy. The patient developed isolated weakness and clawing of the fourth and fifth digits, initially suggestive of an ulnar neuropathy. Distinguishing between a peripheral ulnar nerve lesion and proximal pathology such as cervical radiculopathy or metastatic disease can be challenging in clinical practice [3]. Studies have demonstrated that even experienced clinicians may have difficulty differentiating C8-T1 radiculopathy from ulnar neuropathy based on examination alone, particularly when symptoms are confined to the hand [3]. In this case, the absence of neck pain, radicular pain, or clear upper motor neuron signs further contributed to diagnostic uncertainty. However, MRI subsequently demonstrated extensive metastatic involvement of the cervical vertebrae with neuroforaminal extension and spinal canal stenosis, providing a clear anatomical explanation for the patient’s progressive neurological deficit.

Radiological assessment plays a crucial role in guiding the management of spinal metastatic disease [4]. The epidural spinal cord compression (ESCC) grading system has been developed to standardise MRI evaluation and improve communication between radiologists and clinicians when determining the severity of compression and potential treatment strategies [4]. In our patient, the findings of extensive vertebral metastases, neuroforaminal involvement, and pathological fracture were strongly suggestive of clinically significant cervical spinal disease. In such cases, prompt multidisciplinary evaluation involving oncology, neurosurgery, and spinal teams is essential to determine the most appropriate management approach [2].

Management strategies for MSCC depend on several factors, including neurological status, tumour histology, spinal stability, overall disease burden, and expected survival [2,5]. Evidence from a landmark randomised controlled trial demonstrated that direct decompressive surgery followed by radiotherapy can improve functional outcomes and maintain ambulation compared with radiotherapy alone in selected patients with MSCC [5]. However, surgical intervention is not always appropriate, particularly in patients with advanced metastatic disease, significant frailty, or limited life expectancy [2,5].

Corticosteroids are commonly administered in suspected or confirmed MSCC to reduce tumour-associated oedema and alleviate spinal cord compression [6,7]. Clinical recommendations suggest dexamethasone at a dose of approximately 16 mg per day, balancing the potential neurological benefits with the risks of complications associated with higher doses [6]. Subsequent tapering is typically performed once definitive therapy, such as radiotherapy, has been delivered [7]. In the present case, the patient received high-dose dexamethasone according to an established protocol, alongside cervical immobilisation and supportive care.

Radiotherapy continues to play a central role in the palliative management of metastatic spinal disease, particularly in patients who are not surgical candidates [2,8]. Short-course regimens are frequently used in patients with limited life expectancy in order to minimise treatment burden while providing effective symptom control [8]. Randomised trials have shown that a single fraction of 8 Gy can provide comparable functional outcomes and symptom relief to longer radiotherapy schedules in patients with MSCC and short predicted survival [8]. The patient in this case received single-fraction palliative radiotherapy to the cervical spine, which stabilised the neurological deficit but did not lead to meaningful functional recovery.

This case highlights the importance of maintaining a high index of suspicion for central neurological causes in patients with cancer who develop new focal limb deficits. Apparent peripheral nerve palsies may in fact represent proximal neurological pathology, and reliance on classical features such as back pain or upper motor neuron signs may delay recognition [1-3]. Early spinal imaging and multidisciplinary evaluation are therefore essential to facilitate timely diagnosis, guide management decisions, and optimise symptom control in patients with metastatic disease.

## Conclusions

This case illustrates an atypical presentation of MSCC manifesting as an apparent isolated ulnar nerve palsy with rapid hand clawing. The absence of pain, upper motor neuron signs, or widespread neurological deficits contributed to diagnostic uncertainty. In patients with known malignancy, even subtle or focal neurological symptoms should prompt urgent consideration of central causes and early spinal imaging. This report highlights the risk of diagnostic anchoring and reinforces the importance of maintaining a high index of suspicion for cervical metastatic disease, as timely recognition remains crucial for appropriate management, symptom control, and patient-centred care in advanced cancer.
